# Prospective *Salmonella* Enteritidis surveillance and outbreak detection using whole genome sequencing, Minnesota 2015–2017

**DOI:** 10.1017/S0950268820001272

**Published:** 2020-06-16

**Authors:** J. M. Rounds, A. J. Taylor, D. Eikmeier, M. M. Nichols, V. Lappi, S. E. Wirth, D. J. Boxrud, K. E. Smith, C. Medus

**Affiliations:** 1 Minnesota Department of Health, Saint Paul, Minnesota, USA; 2 New York State Department of Health/Wadsworth Center, Albany, New York, USA

**Keywords:** Food-borne infections, molecular epidemiology, pulsed-field gel electrophoresis, *Salmonella*, surveillance system

## Abstract

Clusters of *Salmonella* Enteritidis cases were identified by the Minnesota Department of Health using both pulsed-field gel electrophoresis (PFGE) and whole genome sequencing (WGS) single nucleotide polymorphism analysis from 1 January 2015 through 31 December 2017. The median turnaround time for obtaining WGS results was 11 days longer than for PFGE (12 *vs.* 1 day). WGS analysis more than doubled the number of clusters compared to PFGE analysis, but reduced the total number of cases included in clusters by 34%. The median cluster size was two cases for WGS compared to four for PFGE, and the median duration of WGS clusters was 27 days shorter than PFGE clusters. While the percentage of PFGE clusters with a confirmed source (46%) was higher than WGS clusters (32%), a higher percentage of cases in clusters that were confirmed as outbreaks reported the vehicle or exposure of interest for WGS (78%) than PFGE (46%). WGS cluster size was a significant predictor of an outbreak source being confirmed. WGS data have enhanced *S*. Enteritidis cluster investigations in Minnesota by improving the specificity of cluster case definitions and has become an integral part of the *S*. Enteritidis surveillance process.

## Introduction

Whole genome sequencing (WGS) as a method for bacterial genomic characterisation is rapidly being implemented into public health foodborne outbreak detection workflows [1–5], and has already shown the ability to retrospectively delineate outbreak and non-outbreak-related *Salmonella enterica* isolates, including serovar Enteritidis, with greater resolution than traditional subtyping methods [6–15].

Prospective use of WGS single nucleotide polymorphism (SNP) analysis in combination with real-time epidemiological investigation for outbreak surveillance is in its infancy, but has shown promise [9, 16–18]. At the Minnesota Department of Health (MDH) Public Health Laboratory (PHL), WGS was routinely performed in parallel with pulsed-field gel electrophoresis (PFGE) subtyping and epidemiological investigation to detect and investigate *S.* Enteritidis clusters beginning in April 2014. All clusters identified by PFGE subtyping and WGS SNP analysis were investigated epidemiologically as potential outbreaks.

Here, we describe the characteristics of the *S.* Enteritidis clusters identified using either PFGE and/or WGS SNP analysis during 2015–2017. We also compare the epidemiological significance and concordance of PFGE and WGS clusters. We demonstrate the utility of an SNP-based analysis subtyping method as a valuable tool for identifying outbreaks and supporting the epidemiological investigation of *S.* Enteritidis clusters of public health importance.

## Materials and methods

### Isolate criteria

All *S.* Enteritidis isolates from human clinical specimens collected from 1 January 2015 through 31 December 2017 and submitted to MDH PHL were included in this study. Also included were closely related antigenic variants of serotype Enteritidis with PFGE patterns matching previously analysed *S*. Enteritidis isolates. Isolates from non-Minnesota residents and non-human isolates were excluded. Duplicate isolates from the same person that were collected within a year of each other were also excluded. See Supplementary Table S1 on the Cambridge Core website for isolate metadata.

### Serotyping and PFGE

All isolates were serotyped phenotypically using the Kaufman–White–Le Minor scheme [19, 20]. All isolates were also subtyped using PFGE with two enzymes, XbaI and BlnI, using standard methods [21]. Patterns were analysed using Bionumerics (Applied Maths) with a Dice coefficient and a 1% band matching criterion. Patterns with no noticeable band differences (presence, absence or noticeable size shift) were considered indistinguishable and assigned PFGE primary (XbaI) and secondary (BlnI) patterns using the MDH pattern designation scheme. Corresponding Centers for Disease Control and Prevention PulseNet USA Network PFGE pattern designations are presented in this paper.

### Whole genome sequencing and SNP typing

WGS of all isolates was performed in as near real-time as resources allowed, using methods previously described [15] with no modifications. An SNP-based comparison analysis was performed by the Wadsworth Center/New York State Department of Health bioinformatics core facility as described previously [15] with no modifications. Turnaround times were calculated in calendar days for isolates that had complete date records. Isolates with turnaround >60 days were excluded (as these indicated exceptional circumstances that did not reflect normal processing times).

Raw sequence data were uploaded in real-time to the National Center for Biotechnology Information (NCBI) Sequence Read Archive (SRA) under BioProject PRJNA237212 (GenomeTrakr real-time SE: Minnesota Department of Health) or PRJNA230403 (PulseNet *Salmonella enterica* Genome sequencing). See Supplementary Table S1 on the Cambridge Core website for isolate SRA accessions. Dendrogram visualisation of newick files produced by the Wadsworth SNP analysis pipeline was created using the Interactive Tree of Life (iTOL) v4 [22].

The discriminatory power for PFGE and WGS was calculated using Simpson's index of diversity [23]. For this calculation, an isolate was assigned the same PFGE type as another isolate when they had the same primary (XbaI) and secondary (BlnI) PFGE pattern combination. An isolate was assigned the same WGS type as another isolate when they were ⩽5 SNPs different. PFGE and WGS cluster comparisons were visualised using an alluvial diagram created R package ‘alluvial’ [24, 25].

### Case reporting and interviewing

Salmonella infections are reportable in Minnesota, and clinical laboratories are required to submit an isolate or specimen from positive clinical samples to the MDH PHL as part of reportable disease rules. All Minnesota residents with a culture-confirmed salmonella infection are interviewed as soon as possible by MDH staff with a standard questionnaire about symptom history, food consumption and other potential exposures occurring in the 7 days before the onset of illness. The questionnaire contains detailed food exposure questions, including open-ended food histories and objective yes/no questions about numerous specific food items, as well as brand names and purchase locations. Clusters are investigated using an iterative model in which suspicious exposures identified during initial cases interviews are added to the standard interview for subsequent cases. Similarly, initial cluster cases may be reinterviewed to ensure uniform ascertainment of the suspicious exposures. This iterative approach is used to identify exposures for further evaluation with formal hypothesis testing, product sampling or product tracing [26, 27].

### Cluster definitions

A PFGE cluster was defined as ⩾2 cases in different households that submitted *S.* Enteritidis isolates with the same XbaI and BlnI PFGE patterns and with specimen collection dates within 60 days. Thus, a single cluster would be ongoing as long as a new isolate matching by PFGE was collected within 60 days after the most recent isolate in the cluster.

A WGS cluster was defined as ⩾2 cases in different households that submitted *S.* Enteritidis isolates with ⩽5 SNP differences and with specimen collection dates within 60 days. Thus, a single cluster would be ongoing as long as a new isolate matching by WGS was collected within 60 days after the most recent isolate in the cluster. A cut-off of five SNPs was chosen based on the SNP diversity observed from a previous retrospective analysis of *S.* Enteritidis outbreaks in Minnesota [15].

Clusters identified either by PFGE pattern analysis and SNP distances were communicated to epidemiologists as soon as each analysis was finished. A cluster, by either method definition, was considered a confirmed outbreak if the epidemiological evaluation of that cluster resulted in the identification of a common source of infection for those cases.

## Results

### Isolate overview and laboratory workflow

From 1 January 2015 through 31 December 2017, the MDH PHL received or recovered 660 *S*. Enteritidis clinical isolates from Minnesota residents. An additional 150 non-human isolates, 35 isolates from non-Minnesota residents and 65 duplicate isolates from the same patient were received at the MDH PHL but were excluded from this study.

**Fig. 1.**
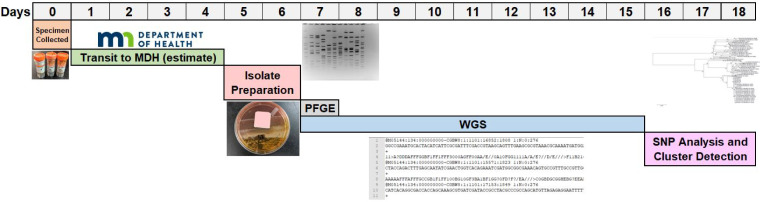
Median times for *Salmonella* Enteritidis surveillance workflow, Minnesota, 2015–2017.

After isolate recovery and/or preparation in the laboratory, isolates were sent simultaneously for serotyping, PFGE and WGS. Turnaround times for PFGE and WGS were calculated for 624 samples (95%). The median turnaround time for obtaining PFGE cluster results from isolate recovery was one calendar day (range 1–7). The median turnaround time for WGS cluster results from isolate recovery was 12 calendar days (range 4–57, 9 days for isolate sequencing and 3 days for SNP analysis and cluster determination) (Fig. 1).

## PFGE clusters

During 2015–2017, there were 39 *S.* Enteritidis PFGE clusters comprising 538 isolates (82% of study isolates). The median number of cases in each cluster was 4 (range, 2–99 cases). The median duration of each cluster was 56 days (range, 2–1043 days). A confirmed source was identified in 18 (46%) clusters. These included eight foodborne outbreaks, eight international travel-associated outbreaks in which multiple cases reported staying at the same resort and two outbreaks associated with live poultry contact. A probable source was identified for five (13%) additional clusters; all five were associated with international travel during which cases reported travelling to the same city. A median of 34% of cases in the clusters with a confirmed source reported the outbreak vehicle or exposure of interest (range, 4–100%). There were three clusters that included cases in two different confirmed outbreaks. For the 10 foodborne and poultry contact-associated outbreaks, a median of 46% of cases in the clusters reported the outbreak vehicle or exposure of interest (range, 4–100%).

The probability of identifying a confirmed source for a PFGE cluster increased significantly as the number of cluster cases increased (*P* = 0.01). Eighty-eight per cent of PFGE clusters of five or more cases had a confirmed source compared to 0% of PFGE clusters of four cases, 50% of clusters of three cases and 7% of clusters of two cases (Table 1).

**Table 1.** Univariate association between *Salmonella* Enteritidis pulsed-field gel electrophoresis (PFGE) and whole genome sequencing (WGS) cluster size and a confirmed or probable source being identified, Minnesota, 2015–2017

Cluster Size	PFGE				Odds ratio^a^^,^*	95% CI^a^	WGS				Odds ratio^a^,*	95% CI^a^
	Confirmed no. (%)	Probable no. (%)	Unsolved no. (%)	Total no.			Confirmed no. (%)	Probable no. (%)	Unsolved no. (%)	Total		
2	1 (7)	2 (14)	11 (79)	14	Ref		6 (13)	7 (15)	35 (73)	48	Ref	
3	2 (50)	0 (0)	2 (50)	4	3.3	0.3–33.4	3 (20)	4 (27)	8 (53)	15	2.3	0.7–7.8
4	0 (0)	2 (50)	2 (50)	4	3.3	0.3–33.4	3 (30)	2 (20)	5 (50)	10	2.6	0.7–10.6
5 +	15 (88)	1 (6)	1 (6)	17	36	4.3–300.9	17 (89)	2 (11)	0 (0)	19	102.6	5.4–undefined
Total	18 (46)	5 (13)	16 (41)	39			29 (32)	15 (16)	48 (52)	92		

a Odds ratio and 95% confidence interval comparing clusters with confirmed and probable source to unsolved clusters and includes Firth correction due to small cell size.

*Significant Mantel–Haenszel *χ*^2^ test for trend *P* = 0.01.

## WGS clusters

During 2015–2017, there were 92 *S.* Enteritidis WGS clusters comprising 356 isolates (54% of study isolates). The median minimum number of SNPs between isolates within a WGS cluster was 1 (range, 0–5 SNPs) and the median maximum number of SNPs between isolates within a cluster was 3 (range, 0–10 SNPs). The median number of cases in each cluster was 2 (range, 2–18 cases). The median duration of each cluster was 29 days (range, 0–247 days). A confirmed source was identified in 29 (32%) clusters. These included 15 international travel-associated outbreaks in which multiple cases reported staying at the same resort, nine foodborne outbreaks and five outbreaks associated with live poultry contact. A probable source was identified for 15 (16%) additional clusters, all associated with international travel during which cases reported travelling to the same city. A median of 67% of cases in the clusters with a confirmed source reported the outbreak vehicle or exposure of interest (range, 20–100%). In contrast to PFGE, no clusters contained cases from multiple independent outbreaks. For the 14 foodborne and animal contact-associated outbreaks, a median of 78% of cases in the clusters reported the outbreak vehicle or exposure of interest (range, 33–100%).

The probability of identifying a confirmed source for a WGS cluster increased significantly as the number of cluster cases increased (*P* = 0.01). Eighty-nine per cent of WGS clusters of five or more cases had a confirmed source compared to 30% of WGS clusters of four cases, 20% of clusters of three cases and 13% of clusters of two cases (Table 1).

## WGS *vs.* PFGE clusters

**Fig. 2.**
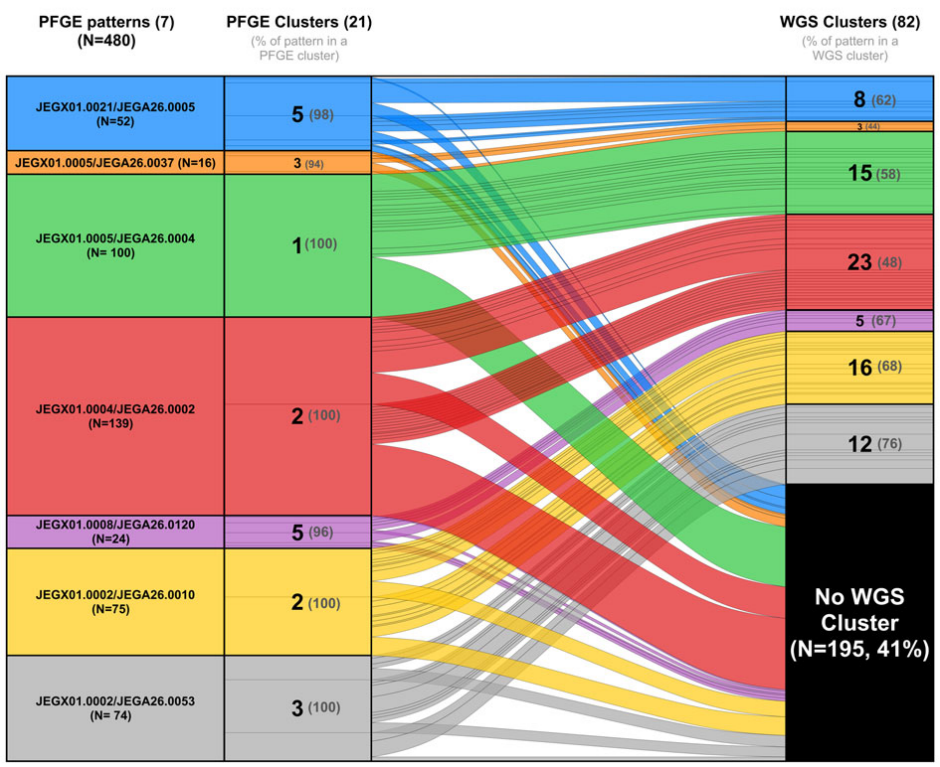
Alluvial diagram comparison of *Salmonella* Enteritidis clusters by PFGE and WGS for the seven most common XbaI/BlnI PFGE pattern combinations during the study, Minnesota, 2015–2017. The ‘PFGE patterns’ column shows the seven most common PFGE pattern combinations during the study period with the size of the coloured block proportional to the pattern's frequency. The ‘PFGE Clusters’ column indicates the number of PFGE clusters of that pattern combination that occurred (the grey numbers in parentheses indicate the percentage of isolates of that PFGE pattern combination that were assigned into a PFGE cluster). The ‘WGS Clusters’ column indicates if the isolates of that PFGE pattern combination grouped into a WGS cluster, and if so, how many WGS clusters were formed and what percentage were assigned to WGS clusters. The coloured lines within each of the PFGE pattern combination boxes for PFGE and WGS clusters outline distinct clusters, with the size of the boxes being proportional to cluster size. For example, 52 isolates in the study were the JEGX01.0021/JEGA26.0005 pattern combination (blue), 98% of which grouped into PFGE clusters, 62% of those isolates grouped into eight WGS clusters and the remainder did not group into a WGS cluster. Note that the WGS clusters formed do not necessarily only contain isolates of these PFGE pattern combinations, but only the isolates from these PFGE pattern combinations are shown.

Six (15%) PFGE clusters contained cases that also belonged to two or more WGS clusters (Fig. 2). Four (4%) WGS clusters contained cases that also belonged to two or more PFGE clusters. Twenty-one (23%) WGS clusters had case isolates with multiple PFGE patterns (XbaI and/or BlnI). The median number of band differences between isolates in the same WGS cluster with different XbaI patterns was one band (range, 1 to 4 bands). The median number of band differences between isolates in the same WGS cluster with different BlnI patterns was two bands (range, 1–15 bands).

The discriminatory power of PFGE using two enzymes was 0.89 and of WGS was 0.99. PFGE clusters contained significantly more cases and had a significantly longer duration than WGS clusters (Table 2). WGS split large and long duration PFGE clusters of isolates with patterns JEGX01.0004 and JEGX01.0002 into a greater number of smaller, shorter duration WGS clusters (Fig. 2 and Table 2).

**Table 2.** *Salmonella* Enteritidis cluster comparison between pulsed-field gel electrophoresis (PFGE) and whole genome sequencing (WGS), Minnesota, 2015–2017

	PFGE	WGS	*P* value^a^
No. of clusters	39	92	
No. cluster isolates	538	356	
No. (%) clusters with confirmed source	18 (46%)	29 (32%)	
% of cases in outbreak cluster that report outbreak vehicle (range)	34% (4–100)	67% (20–100)	
Median no. cases per cluster (range)	4 (2–99)	2 (2–18)	0.018
Median no. days duration of cluster (range)	56 (2–1043)	29 (0–247)	0.001
No. (%) clusters with ⩾10 cases	12 (31%)	7 (8%)	
No. clusters with PFGE pattern JEGX01.0004/JEGA26.0002 isolates	2	23	
No. clusters with PFGE pattern JEGX01.0002/JEGA26.0010 isolates	2	15	

a Kruskal–Wallis test.

## Outbreak investigation examples

### Stuffed chicken-associated outbreak within a background of numerous sporadic cases with a common PFGE pattern

**Fig. 3.**
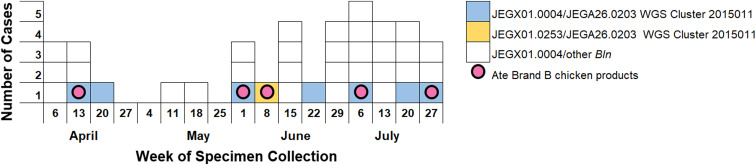
*Salmonella* Enteritidis JEX01.0004 and JEGX01.0253 clinical cases by secondary pulsed-field gel electrophoresis enzyme (BlnI), whole genome sequencing cluster status, exposure to implicated outbreak vehicle and week of specimen collection, Minnesota, April–July 2015.

From April through July 2015, MDH PHL identified 37 *S.* Enteritidis isolates with the primary enzyme (XbaI) PFGE pattern JEX01.0004 (Fig. 3). Five (14%) cases reported eating the same brand of stuffed chicken product (Brand B), suggesting a common source outbreak. However, determining whether the pattern JEX01.0004 cases that denied consuming that product were associated with the outbreak was difficult. WGS results determined that eight cases were closely related to each other (0–2 SNPs, cluster 2015011), including one case with an XbaI pattern (JEGX01.0253) that was one band different from JEX01.0004. All isolates in the WGS cluster also had the same second enzyme (BlnI) PFGE pattern (JEGA26.0203). Brand B stuffed chicken product collected from case households and from retail establishments tested positive for the *S.* Enteritidis outbreak PFGE pattern JEX01.0004/JEGA26.0203 and isolates were 0–2 SNPs from the human case isolates by WGS. This investigation ultimately identified 15 cases in seven states [28]. WGS allowed investigators to exclude other *S.* Enteritidis pattern JEX01.0004 cases and to include a case with a different primary enzyme PFGE pattern. This outbreak demonstrated the utility of WGS in increasing the specificity of the case definition for *S.* Enteritidis clusters.

### Concurrent independent outbreaks among cases with the same common PFGE pattern

**Fig. 4.**
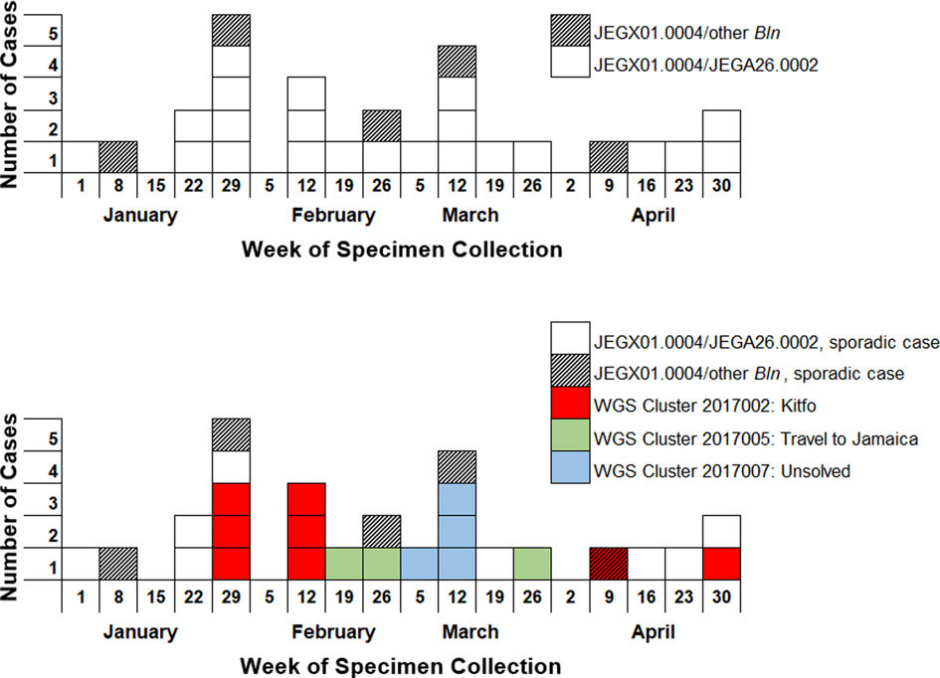
*Salmonella* Enteridis pulsed-field gel electrophoresis (PFGE) pattern JEGX01.0004 clinical cases in Minnesota by week of specimen collection, January–April 2017. In the top graph, primary (XbaI) and secondary (BlnI) PFGE patterns are marked as indicated. In the bottom graph, clusters with a source confirmed by epidemiological investigation are coloured as indicated. The WGS cluster 2017002 cases from the weeks of April 9 and April 30 were not considered part of the kitfo outbreak for several reasons: the isolates were two SNPs from the kitfo case isolates which were all zero SNPs from each other; they occurred almost 2 months after the outbreak; they had different demographics than the kitfo cases; and they did not report consuming kitfo.

**Fig. 5.**
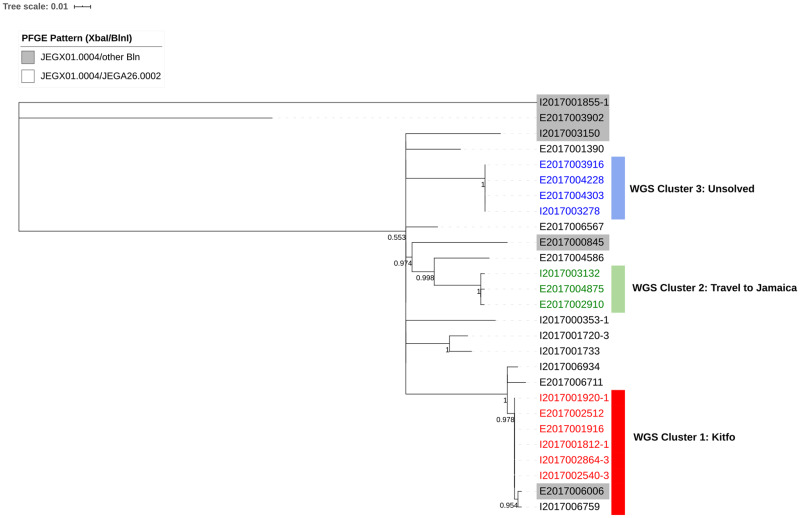
Maximum-likelihood tree produced by single nucleotide polymorphism (SNP) analysis of sequences of *Salmonella* Enteridis clinical isolates collected in Minnesota, January–April 2017. Unshaded isolates are pulsed-field gel electrophoresis (PFGE) pattern JEGX01.0004/JEGA26.0002, and shaded isolates are PFGE pattern JEGX01.0004/other *Bln*. Whole genome sequencing (WGS) clusters are indicated by coloured bars, and confirmed clusters by epidemiological investigation are indicated by coloured isolate identifiers. Values at the base of the nodes are approximate likelihood-ratio test values. The scale bar indicates substitutions per site.

MDH PHL received 27 clinical isolates of *S.* Enteritidis with the PFGE pattern JEGX01.0004 from 1 January through 3 May 2017 (Fig. 4). The use of the secondary PFGE restriction enzyme (BlnI) provided limited additional discrimination in this instance; the majority of the isolates (*n* = 22, 81%) were JEGA26.0002 (Fig. 4). WGS revealed three distinct clusters of 8, 4 and 3 isolates, and 12 unrelated isolates (Figs 4 and 5) within this PFGE cluster. Epidemiological investigation of WGS cluster 2017002 (*n* = 8) identified an outbreak associated with the consumption of kitfo, which was made with undercooked ground beef from a grocery store. While the first six cases in this cluster all reported consuming kitfo and were zero SNPs from each other, the last two cases in this cluster were two SNPs from the other cases, did not fit the demographics of the kitfo outbreak cases, did not report consuming kitfo, and occurred 57 and 76 days later, so they were not considered part of the outbreak. WGS cluster 2017005 (*n* = 3) was associated with travel to a resort in Jamaica, and no source was identified for WGS cluster 2017007 (*n* = 4), although one case did report international travel. The increased case definition specificity provided by WGS helped confirm the scope of the outbreaks.

## Discussion

This prospective study demonstrated that WGS is a more discriminatory and useful subtyping method than PFGE for the detection and investigation of *S*. Enteritidis outbreaks. The proportion of WGS clusters that had a confirmed or probable source identified (48%) was lower than for PFGE clusters (59%). However, WGS detected more than twice as many clusters as PFGE, and this translated into a much higher absolute number of WGS clusters that had a confirmed or probable source identified (*n* = 44) than was true for PFGE (*n* = 23). The benefit of WGS in solving additional outbreaks was seen mostly with clusters of less than five cases.

In addition to providing more sensitive outbreak detection, the increased specificity provided by WGS for cluster case definitions also made investigations more efficient. WGS reduced the total number of cases included in clusters (and thus warranting focused investigation) by 34%, and clusters were smaller on average. In addition, the median duration of WGS clusters was significantly shorter than PFGE clusters, decreasing the likelihood of drawn-out, ongoing investigations with no source ever identified. Finally, a much higher percentage of cases in confirmed outbreaks reported the vehicle or exposure of interest if they belonged to a WGS cluster (78%) *vs.* a PFGE cluster (46%).

The MDH has had success in detecting *S*. Enteritidis outbreaks by PFGE using two enzymes (XbaI and BlnI) for subtyping [27, 29]. However, the current study showed that WGS provided even more discriminatory power than the use of two enzymes. In essence, the greatest value of WGS was that it identified smaller groups of related outbreak cases within a background sea of unrelated sporadic cases that had the same clonal PFGE pattern combinations, and that coincidentally clustered in time because they occurred so commonly.

A challenge of WGS is the interpretation of analysis results, and that the communication between laboratorians and epidemiologists is not as straight-forward as with traditional typing methods that have stable nomenclature, like PFGE. A single SNP distance cut-off was assigned in this study to systematically group isolates and thus inform guidelines for inclusion/exclusion of phylogenetically similar cases in an outbreak investigation. In practice, the SNP cut-off was not strictly adhered to during epidemiological investigations; isolates that fell outside the SNP cut-off were evaluated by epidemiologists to determine if they should be included in the cluster. Epidemiological context was necessary for interpreting SNP typing results, as different outbreak timeframes and transmission types (point source *vs.* polyclonal) present different phylogenetic patterns. Communication between the laboratory, bioinformaticians and epidemiologists was essential in decisions to include or exclude isolates in an outbreak cluster. Nationally, PulseNet USA is using whole genome MLST analysis using allele codes for salmonella foodborne outbreak cluster detection [30]. This provides a standardised analysis method and improves cluster-related communication, especially in comparison to methods that do not provide stable nomenclature.

The practicality of WGS for prospective surveillance is also hindered by turn-around time constraints. The 12-day average turnaround time for sequencing analysis of samples in this study includes multiple days for DNA extraction, library preparation and sequencing due to limited personnel and resources. Additionally, batching samples to meet the most cost-effective capacity of high-throughput short read sequencing instruments increased turn-around time. The delays in the wet-lab protocol, combined with the more computationally intensive analysis requirements of WGS, created delays in epidemiologists receiving actionable WGS results. The national transition of salmonella outbreak surveillance to WGS has allowed more resources to be shifted towards WGS, which has increased timeliness in Minnesota since this study timeframe. Turnaround times will not be fully optimised until the volume of samples is consistently high enough to sequence isolates immediately upon submission, or until the adoption of a sequencing platform capable of handling smaller numbers of samples with reduced cost detriment.

Despite these limitations, WGS data have enhanced *S.* Enteritidis cluster investigations in Minnesota by improving the specificity of cluster case definitions and has become an integral part of the *S.* Enteritidis surveillance process.

## Supplementary material

For supplementary material accompanying this paper visit http://dx.doi.org/10.1017/S0950268820001272. click here to view supplementary material
