# Supplementary material for: Prospective Salmonella Enteritidis surveillance and outbreak detection using whole genome sequencing, Minnesota 2015–2017
Source: Epidemiol Infect. 2020 Jun 16;148:e254. doi: 10.1017/S0950268820001272 (PMC7689598; doi:10.1017/S0950268820001272)
Supplement: Supplementary file 1 [file S0950268820001272sup001.docx]

**Epidemiology and Infection**

**Title:** Prospective *Salmonella* Enteritidis Surveillance and Outbreak Detection using Whole Genome Sequencing, Minnesota 2015-2017

J. M. Rounds*^a^, A. J. Taylor*^a^**,**  D. Eikmeier^a^, M. M. Nichols^a^, V. Lappi^a^, S. E. Wirth^b^ , D. J. Boxrud^a^, K. E. Smith^a^, C. Medus^a^

***Co-first authors**

**Author affiliations:** Minnesota Department of Health, Saint Paul, Minnesota, USA^a^; New York State Department of Health/Wadsworth Center, Albany, New York, USA^b^

Supplementary Material

**Supplementary Table S1: Isolate metadata**

| **Minnesota Department of Health Identifier** | **Collection Date** | **PFGE Cluster Code** | **WGS Cluster Code** | **NCBI SRA Accession** |
| --- | --- | --- | --- | --- |
| E2015000482 | 1/7/2015 | 1 |  | SRR1765787 |
| E2015000449 | 1/7/2015 |  |  | SRR1774018 |
| E2015000899 | 1/13/2015 | 1 |  | SRR1774023 |
| E2015000928 | 1/13/2015 | 1 |  | SRR1828970 |
| E2015001003 | 1/13/2015 | 1 |  | SRR1828968 |
| E2015001410 | 1/21/2015 | 1 |  | SRR1998948 |
| E2015001449 | 1/24/2015 | 1 | 2015006 | SRR2035401 |
| E2015001636 | 1/28/2015 |  |  | SRR2062504 |
| E2015002750 | 2/17/2015 | 1 | 2015006 | SRR2088733 |
| E2015002751 | 2/17/2015 |  |  | SRR2102420 |
| E2015002780 | 2/19/2015 | 1 |  | SRR2106118 |
| E2015003054 | 2/23/2015 | 1 | 2015006 | SRR2153465 |
| E2015004392 | 3/25/2015 | 1 |  | SRR2584381 |
| E2015004977 | 4/6/2015 | 1 | 2015010 | SRR2993269 |
| E2015005015 | 4/7/2015 | 1 |  | SRR3173548 |
| E2015005180 | 4/11/2015 | 1 | 2015010 | SRR3490025 |
| E2015005321 | 4/13/2015 | 5 | 2015011 | SRR3490020 |
| E2015006294 | 4/14/2015 | 1 |  | SRR3571281 |
| E2015005610 | 4/17/2015 | 3 | 2015014 | SRR3696018 |
| E2015005617 | 4/21/2015 | 5 | 2015011 | SRR3743174 |
| E2015006261 | 5/11/2015 | 3 | 2015014 | SRR4095256 |
| E2015006897 | 5/19/2015 | 1 |  | SRR4256615 |
| E2015007246 | 6/2/2015 | 1 |  | SRR5000320 |
| E2015007367 | 6/3/2015 | 1 |  | SRR5217786 |
| E2015007293 | 6/3/2015 | 5 | 2015011 | SRR5361028 |
| E2015008162 | 6/16/2015 | 1 | 2015019 | SRR5366226 |
| E2015008222 | 6/18/2015 | 1 | 2015019 | SRR5408969 |
| E2015008277 | 6/20/2015 | 3 |  | SRR5408953 |
| E2015008299 | 6/21/2015 | 1 | 2015026 | SRR5413701 |
| E2015008461 | 6/24/2015 | 5 | 2015011 | SRR5440656 |
| E2015008686 | 6/30/2015 | 1 |  | SRR5583957 |
| E2015008788 | 7/2/2015 | 1 |  | SRR5590195 |
| E2015008712 | 7/3/2015 | 1 | 2015023 | SRR5851117 |
| E2015009163 | 7/4/2015 | 1 |  | SRR5808903 |
| E2015009250 | 7/8/2015 | 1 |  | SRR5851138 |
| E2015009381 | 7/8/2015 | 1 |  | SRR5864655 |
| E2015009338 | 7/8/2015 | 1 | 2015027 | SRR5920086 |
| E2015009961 | 7/10/2015 | 1 | 2015023 | SRR5947626 |
| E2015009530 | 7/11/2015 | 5 | 2015011 | SRR5947618 |
| E2015009730 | 7/13/2015 | 1 |  | SRR5951446 |
| E2015009582 | 7/13/2015 | 3 |  | SRR5947656 |
| E2015009798 | 7/15/2015 | 1 | 2015022 | SRR6029163 |
| E2015009813 | 7/16/2015 | 1 | 2015032 | SRR6019678 |
| E2015010289 | 7/21/2015 | 1 | 2015026 | SRR6029181 |
| E2015010245 | 7/22/2015 | 1 |  | SRR10403303 |
| E2015010228 | 7/23/2015 | 1 |  | SRR6025999 |
| E2015010334 | 7/24/2015 | 1 | 2015027 | SRR6038714 |
| E2015010527 | 7/24/2015 | 5 | 2015011 | SRR6107284 |
| E2015010902 | 7/29/2015 | 1 |  | SRR6029179 |
| E2015010635 | 7/29/2015 | 5 | 2015011 | SRR6039024 |
| E2015011911 | 8/19/2015 |  | 2015031 | SRR6396466 |
| E2015012194 | 8/26/2015 |  |  | SRR1822279 |
| E2015012441 | 8/31/2015 |  |  | SRR1982119 |
| E2015012647 | 9/3/2015 | 1 | 2015032 | SRR5105393 |
| E2015012817 | 9/3/2015 | 3 |  | SRR5105397 |
| E2015013177 | 9/13/2015 | 3 | 2015033 | SRR5947635 |
| E2015014162 | 9/17/2015 | 1 |  | SRR1765786 |
| E2015013787 | 9/23/2015 | 1 |  | SRR1773593 |
| E2015014267 | 10/2/2015 | 1 |  | SRR1982154 |
| E2015014863 | 10/11/2015 | 1 |  | SRR1988470 |
| E2015014948 | 10/16/2015 | 3 | 2015033 | SRR1988476 |
| E2015015240 | 10/19/2015 | 1 |  | SRR1988452 |
| E2015015259 | 10/20/2015 | 1 |  | SRR1988491 |
| E2015015342 | 10/22/2015 |  |  | SRR1982197 |
| E2015017252 | 11/21/2015 | 1 |  | SRR2035406 |
| E2015017577 | 12/5/2015 |  |  | SRR2054257 |
| E2015017909 | 12/9/2015 | 1 |  | SRR2056000 |
| E2016000456 | 12/31/2015 | 1 |  | SRR2175357 |
| E2016001974 | 2/1/2016 | 1 |  | SRR3173546 |
| E2016002711 | 2/17/2016 | 1 |  | SRR3198635 |
| I2016002520 | 2/18/2016 | 1 | 2016009 | SRR3210396 |
| E2016004291 | 3/20/2016 | 1 | 2016009 | SRR3400296 |
| E2016005001 | 3/31/2016 | 1 | 2016014 | SRR3696034 |
| E2016005325 | 4/5/2016 | 1 |  | SRR5182251 |
| I2016005701 | 4/17/2016 | 1 |  | SRR5585203 |
| E2016005845 | 4/19/2016 | 1 | 2016014 | SRR5413690 |
| I2016006732 | 5/5/2016 | 1 |  | SRR5584546 |
| E2016008173 | 5/25/2016 | 1 | 2016020 | SRR5947639 |
| I2016007916 | 5/25/2016 | 1 |  | SRR5993006 |
| I2016008251 | 5/31/2016 | 1 |  | SRR6038722 |
| I2016008677 | 6/9/2016 | 1 | 2016020 | SRR6425499 |
| E2016009469 | 6/21/2016 |  |  | SRR5105395 |
| E2016009483 | 6/22/2016 |  |  | SRR1982155 |
| E2016009765 | 6/23/2016 |  | 2016021 | SRR5749998 |
| E2016010134 | 6/30/2016 | 1 |  | SRR3571280 |
| E2016010175 | 7/2/2016 | 4 |  | SRR3743172 |
| E2016010254 | 7/4/2016 | 4 |  | SRR3634510 |
| E2016011020 | 7/18/2016 | 1 |  | SRR5486493 |
| E2016011044 | 7/19/2016 | 1 |  | SRR5486491 |
| I2016011233 | 7/21/2016 | 1 | 2016036 | SRR5486506 |
| I2016011230 | 7/22/2016 | 1 | 2016036 | SRR5583968 |
| E2016011826 | 8/2/2016 | 1 | 2016030 | SRR1982142 |
| E2016012117 | 8/5/2016 | 1 |  | SRR2914508 |
| E2016012932 | 8/18/2016 | 1 |  | SRR5105403 |
| E2016013647 | 8/29/2016 | 1 |  | SRR5360958 |
| E2016013694 | 8/29/2016 | 1 |  | SRR10403304 |
| I2016013863 | 9/1/2016 | 1 |  | SRR6221665 |
| E2016013873 | 9/2/2016 | 1 | 2016029 | SRR6300186 |
| I2016013970 | 9/7/2016 | 1 |  | SRR6300173 |
| E2016014886 | 9/9/2016 | 1 | 2016029 | SRR6327001 |
| I2016014362 | 9/11/2016 | 1 |  | SRR6371533 |
| I2016014504 | 9/13/2016 | 1 | 2016030 | SRR2153208 |
| I2016016334 | 10/14/2016 | 1 |  | SRR5501020 |
| E2016016679 | 10/20/2016 | 1 |  | SRR3990984 |
| E2016017033 | 10/27/2016 | 1 |  | SRR1828979 |
| I2017000353 | 1/5/2017 | 2 |  | SRR2088736 |
| E2017000845 | 1/11/2017 |  |  | SRR5920143 |
| E2017001390 | 1/23/2017 | 2 |  | SRR3372231 |
| I2017001733 | 1/28/2017 | 2 |  | SRR5501016 |
| I2017001720 | 1/30/2017 | 2 |  | SRR3509913 |
| I2017001812 | 2/1/2017 | 2 | 2017002 | SRR3509907 |
| I2017001920 | 2/1/2017 | 2 | 2017002 | SRR3490012 |
| I2017001855 | 2/1/2017 |  |  | SRR3490015 |
| E2017001916 | 2/2/2017 | 2 | 2017002 | SRR3824043 |
| E2017002512 | 2/12/2017 | 2 | 2017002 | SRR5360990 |
| I2017002864 | 2/15/2017 | 2 | 2017002 | SRR5947657 |
| I2017002540 | 2/16/2017 | 2 | 2017002 | SRR6026003 |
| E2017002910 | 2/20/2017 | 2 | 2017005 | SRR5920101 |
| I2017003132 | 2/27/2017 | 2 | 2017005 | SRR1773839 |
| I2017003150 | 3/3/2017 |  |  | SRR1828969 |
| I2017003278 | 3/6/2017 | 2 | 2017007 | SRR1822281 |
| E2017003916 | 3/13/2017 | 2 | 2017007 | SRR2047357 |
| E2017003902 | 3/14/2017 |  |  | SRR2035408 |
| E2017004228 | 3/15/2017 | 2 | 2017007 | SRR2054262 |
| E2017004303 | 3/16/2017 | 2 | 2017007 | SRR2047361 |
| E2017004586 | 3/23/2017 | 2 |  | SRR2088726 |
| E2017004875 | 3/27/2017 | 2 | 2017005 | SRR2153468 |
| E2017006006 | 4/14/2017 |  | 2017002 | SRR2906165 |
| E2017006711 | 4/20/2017 | 2 |  | SRR3110498 |
| E2017006567 | 4/27/2017 | 2 |  | SRR3184144 |
| I2017006934 | 5/2/2017 | 2 | 2017016 | SRR3330236 |
| I2017006759 | 5/3/2017 | 2 | 2017002 | SRR3400015 |
| I2017008743 | 6/4/2017 | 2 | 2017016 | SRR4022299 |
| I2017008747 | 6/5/2017 | 2 | 2017016 | SRR4022301 |
| I2017008990 | 6/7/2017 | 2 |  | SRR4175552 |
| E2017009257 | 6/13/2017 | 2 |  | SRR4175554 |
| E2017009563 | 6/18/2017 | 2 | 2017002 | SRR4175553 |
| I2017009581 | 6/21/2017 | 6 | 2017002 | SRR5382671 |
| E2017010260 | 6/27/2017 | 2 | 2017016 | SRR5808909 |
| I2017010225 | 6/29/2017 | 2 |  | SRR5822043 |
| I2017010838 | 6/30/2017 | 2 | 2017016 | SRR5823766 |
| I2017011559 | 7/18/2017 |  | 2017016 | SRR6107193 |
| E2017011634 | 7/20/2017 | 2 |  | SRR6330499 |
| E2017011737 | 7/21/2017 | 2 |  | SRR6221659 |
| E2017011877 | 7/21/2017 | 2 | 2017002 | SRR6300180 |
| E2017012408 | 7/31/2017 | 2 | 2017020 | SRR3372227 |
| I2017012478 | 8/2/2017 | 2 |  | SRR3490021 |
| I2017013143 | 8/14/2017 |  |  | SRR4095259 |
| I2017013498 | 8/15/2017 | 2 | 2017021 | SRR1828985 |
| I2017013516 | 8/17/2017 | 2 | 2017019 | SRR1982180 |
| I2017013482 | 8/17/2017 | 6 | 2017002 | SRR1982156 |
| I2017013665 | 8/21/2017 | 2 | 2017019 | SRR2906162 |
| I2017013700 | 8/21/2017 | 2 | 2017020 | SRR2153475 |
| I2017013769 | 8/22/2017 | 2 |  | SRR2532747 |
| E2017014893 | 8/23/2017 | 2 | 2017016 | SRR3037415 |
| E2017014213 | 8/23/2017 | 2 |  | SRR3271880 |
| I2017014274 | 8/25/2017 | 2 |  | SRR3330240 |
| I2017014221 | 8/26/2017 | 2 | 2017002 | SRR3490016 |
| E2017014526 | 8/29/2017 | 2 | 2017002 | SRR3571301 |
| I2017014455 | 8/30/2017 | 2 | 2017021 | SRR3634508 |
| I2017014539 | 8/31/2017 | 2 | 2017021 | SRR3707420 |
| I2017014890 | 9/6/2017 | 2 |  | SRR3824035 |
| E2017015620 | 9/14/2017 | 2 | 2017002 | SRR4175547 |
| I2017015327 | 9/15/2017 | 2 | 2017002 | SRR4237885 |
| I2017015717 | 9/19/2017 | 2 | 2017002 | SRR4427814 |
| E2017015914 | 9/19/2017 | 2 |  | SRR5000201 |
| E2017016447 | 9/27/2017 | 2 |  | SRR5408967 |
| E2017016960 | 10/5/2017 | 2 | 2017002 | SRR5486499 |
| E2017017086 | 10/10/2017 | 2 | 2017026 | SRR5440665 |
| E2017017633 | 10/18/2017 | 2 | 2017024 | SRR5501023 |
| E2017018248 | 10/24/2017 | 2 | 2017024 | SRR5486509 |
| E2017018838 | 11/10/2017 | 2 | 2017026 | SRR6107194 |
| E2017019228 | 11/17/2017 | 2 |  | SRR6195104 |
| I2017019715 | 11/28/2017 |  |  | SRR1982184 |
| I2017020595 | 12/14/2017 | 2 |  | SRR3372225 |
| E2017020894 | 12/21/2017 | 2 |  | SRR5584673 |
| E2018000074 | 12/29/2017 |  |  | SRR2831443 |
| E2015002579 | 2/16/2015 | 9 |  | SRR2088725 |
| E2015003868 | 3/12/2015 | 9 |  | SRR2140787 |
| E2015008481 | 6/13/2015 |  | 2015022 | SRR5366194 |
| E2016007564 | 5/18/2016 | 7 |  | SRR5823589 |
| I2016010304 | 7/6/2016 |  |  | SRR5486469 |
| I2016010355 | 7/9/2016 | 7 |  | SRR5486496 |
| E2016018384 | 11/28/2016 | 10 | 2016031 | SRR5714270 |
| E2016018702 | 11/30/2016 | 10 | 2016031 | SRR1982046 |
| E2017004504 | 3/9/2017 |  |  | SRR2035405 |
| E2017009788 | 6/20/2017 | 8 | 2017019 | SRR5105394 |
| E2017012351 | 7/30/2017 | 8 |  | SRR3330233 |
| E2017014881 | 9/3/2017 | 8 |  | SRR3707419 |
| E2015000393 | 1/6/2015 | 11 | 2015001 | SRR1765792 |
| E2015000947 | 1/10/2015 | 11 | 2015001 | SRR1805601 |
| E2015000930 | 1/14/2015 | 16 |  | SRR1828971 |
| E2015001038 | 1/15/2015 | 11 |  | SRR1822285 |
| E2015001477 | 1/21/2015 | 16 |  | SRR1998979 |
| E2015003007 | 2/19/2015 | 11 | 2015001 | SRR2102471 |
| E2015003208 | 2/24/2015 | 11 | 2015001 | SRR2153466 |
| E2015003578 | 3/4/2015 | 11 | 2015001 | SRR2133171 |
| E2015003585 | 3/4/2015 |  |  | SRR2133235 |
| E2015004088 | 3/8/2015 | 11 | 2015001 | SRR2140788 |
| E2015003889 | 3/15/2015 | 11 | 2015001 | SRR2140789 |
| E2015004070 | 3/18/2015 | 11 | 2015001 | SRR2153205 |
| E2015004044 | 3/19/2015 | 16 |  | SRR2175334 |
| E2015004208 | 3/20/2015 | 16 |  | SRR2533497 |
| E2015004401 | 3/23/2015 | 16 | 2015008 | SRR2533493 |
| E2015004250 | 3/24/2015 | 11 | 2015009 | SRR2533573 |
| E2015004447 | 3/28/2015 | 11 | 2015001 | SRR2724318 |
| E2015004475 | 3/30/2015 | 11 | 2015009 | SRR2724329 |
| E2015004981 | 4/2/2015 | 16 | 2015008 | SRR2906163 |
| E2015005008 | 4/2/2015 | 16 | 2015008 | SRR2906164 |
| E2015005009 | 4/2/2015 | 16 | 2015008 | SRR2906166 |
| E2015004948 | 4/6/2015 | 16 | 2015008 | SRR3037416 |
| E2015004996 | 4/6/2015 | 16 | 2015008 | SRR3110336 |
| E2015005033 | 4/7/2015 | 11 |  | SRR3217344 |
| E2015005181 | 4/10/2015 | 11 | 2015009 | SRR3330232 |
| E2015005672 | 4/23/2015 | 11 | 2015009 | SRR3707421 |
| E2015005681 | 4/23/2015 | 16 |  | SRR3824031 |
| E2015006029 | 5/3/2015 | 16 | 2015008 | SRR3948445 |
| E2015006149 | 5/6/2015 | 11 | 2015001 | SRR3990987 |
| E2015006279 | 5/10/2015 | 11 | 2015001 | SRR4039115 |
| E2015006737 | 5/16/2015 | 11 | 2015009 | SRR4256755 |
| E2015007078 | 5/26/2015 | 11 |  | SRR4256614 |
| E2015007127 | 5/27/2015 | 11 | 2015009 | SRR4294794 |
| E2015007968 | 6/11/2015 | 16 | 2015018 | SRR5281990 |
| E2015008119 | 6/13/2015 |  | 2015018 | SRR5364222 |
| E2015010937 | 8/5/2015 | 16 | 2015035 | SRR6107308 |
| E2015012021 | 8/22/2015 |  |  | SRR6434211 |
| E2015012669 | 9/1/2015 | 12 |  | SRR2088737 |
| E2015013122 | 9/9/2015 | 16 | 2015035 | SRR5851109 |
| E2015013909 | 9/27/2015 | 12 | 2015036 | SRR1828977 |
| E2015014140 | 9/29/2015 | 16 | 2015035 | SRR1982123 |
| E2015014301 | 10/1/2015 | 16 | 2015039 | SRR1982157 |
| E2015014785 | 10/9/2015 |  |  | SRR1988503 |
| E2015015718 | 10/27/2015 | 12 | 2015036 | SRR1988492 |
| E2015017571 | 11/30/2015 | 16 | 2015039 | SRR2035443 |
| E2015017856 | 12/9/2015 | 12 | 2015036 | SRR2056001 |
| E2016000287 | 12/31/2015 | 16 | 2016007 | SRR2533498 |
| E2016000176 | 12/31/2015 | 16 | 2015039 | SRR2533496 |
| E2016001071 | 1/22/2016 | 12 |  | SRR2914510 |
| E2016001531 | 1/25/2016 | 16 | 2016004 | SRR3110501 |
| E2016001533 | 1/27/2016 | 16 | 2016004 | SRR3110499 |
| E2016001746 | 1/29/2016 | 16 | 2016004 | SRR3136811 |
| E2016001727 | 1/30/2016 | 12 | 2016005 | SRR3151855 |
| E2016001927 | 2/3/2016 | 16 | 2016007 | SRR3173545 |
| I2016002162 | 2/9/2016 |  | 2016004 | SRR3242191 |
| E2016002341 | 2/10/2016 | 12 | 2016008 | SRR3188269 |
| E2016002658 | 2/12/2016 | 16 | 2016004 | SRR3210395 |
| E2016002268 | 2/12/2016 | 16 | 2016004 | SRR3184147 |
| E2016002547 | 2/16/2016 | 12 | 2016005 | SRR3217343 |
| E2016002593 | 2/16/2016 | 12 | 2016006 | SRR3198634 |
| E2016002583 | 2/18/2016 | 12 | 2016006 | SRR3217352 |
| E2016002591 | 2/18/2016 | 12 |  | SRR3217345 |
| E2016002697 | 2/20/2016 | 12 |  | SRR3217349 |
| I2016002671 | 2/20/2016 | 16 |  | SRR3217348 |
| E2016002728 | 2/22/2016 | 12 |  | SRR3223811 |
| E2016002889 | 2/25/2016 | 16 |  | SRR3223810 |
| E2016002887 | 2/25/2016 |  |  | SRR3223812 |
| E2016003239 | 2/26/2016 | 12 | 2016008 | SRR3223815 |
| E2016003179 | 2/26/2016 | 16 | 2016007 | SRR3223816 |
| E2016003221 | 2/28/2016 | 12 | 2016008 | SRR3223814 |
| E2016003423 | 2/29/2016 | 12 |  | SRR3223817 |
| E2016003457 | 3/2/2016 | 12 | 2016008 | SRR3383901 |
| E2016003547 | 3/2/2016 |  | 2016008 | SRR3306268 |
| E2016003446 | 3/3/2016 | 16 | 2016004 | SRR3306271 |
| E2016003571 | 3/7/2016 | 12 | 2016008 | SRR3330239 |
| I2016003741 | 3/11/2016 | 16 |  | SRR3383903 |
| E2016003967 | 3/15/2016 | 16 | 2016007 | SRR3383902 |
| E2016004273 | 3/16/2016 |  |  | SRR3372228 |
| E2016004270 | 3/19/2016 | 12 | 2016010 | SRR3400291 |
| E2016004541 | 3/22/2016 | 16 | 2016016 | SRR3400292 |
| I2016004374 | 3/23/2016 | 12 | 2016010 | SRR3400295 |
| E2016004642 | 3/25/2016 | 12 | 2016008 | SRR3465485 |
| E2016004634 | 3/25/2016 | 12 | 2016010 | SRR3490022 |
| I2016004602 | 3/26/2016 | 12 | 2016008 | SRR3622483 |
| I2016004601 | 3/27/2016 | 12 | 2016008 | SRR3571302 |
| E2016004845 | 3/30/2016 | 12 | 2016017 | SRR3634509 |
| E2016004853 | 3/31/2016 | 16 | 2016002 | SRR3824036 |
| E2016005028 | 3/31/2016 | 16 |  | SRR3928752 |
| I2016005030 | 4/2/2016 | 12 | 2016008 | SRR4048576 |
| E2016005076 | 4/3/2016 | 12 | 2016010 | SRR5105396 |
| I2016005047 | 4/3/2016 | 12 | 2016010 | SRR5105398 |
| E2016005293 | 4/4/2016 | 12 | 2016008 | SRR5155666 |
| I2016005110 | 4/5/2016 | 12 | 2016010 | SRR5221478 |
| E2016005458 | 4/9/2016 | 12 | 2016010 | SRR5221484 |
| E2016005628 | 4/11/2016 | 12 | 2016008 | SRR5281995 |
| E2016005692 | 4/12/2016 | 12 | 2016010 | SRR5360713 |
| E2016005950 | 4/21/2016 | 12 |  | SRR5583930 |
| E2016006266 | 4/25/2016 | 16 | 2016016 | SRR5501014 |
| E2016007407 | 4/25/2016 | 16 |  | SRR5440662 |
| E2016006696 | 4/30/2016 | 16 | 2016016 | SRR5501021 |
| E2016007253 | 5/13/2016 | 12 | 2016017 | SRR5583900 |
| I2016007594 | 5/21/2016 | 16 |  | SRR5864530 |
| E2016008211 | 5/28/2016 | 16 |  | SRR5951460 |
| E2016008445 | 6/2/2016 |  | 2016016 | SRR6195119 |
| E2016009850 | 6/25/2016 | 12 | 2016025 | SRR1982188 |
| E2016010140 | 6/28/2016 | 16 |  | SRR1828978 |
| I2016011392 | 7/28/2016 |  |  | SRR5680485 |
| I2016011767 | 8/2/2016 |  |  | SRR2831448 |
| E2016012366 | 8/11/2016 | 12 | 2016025 | SRR3400293 |
| I2016017987 | 11/16/2016 | 13 | 2016033 | SRR6029164 |
| I2016017986 | 11/16/2016 | 13 | 2016033 | SRR4095258 |
| I2016019079 | 12/12/2016 | 13 |  | SRR1828967 |
| E2016019323 | 12/13/2016 | 13 | 2017028 | SRR2035447 |
| E2016019695 | 12/18/2016 | 13 | 2016033 | SRR2054221 |
| I2017000398 | 1/6/2017 | 13 |  | SRR2533494 |
| I2017000384 | 1/7/2017 | 13 |  | SRR2993267 |
| I2017000475 | 1/9/2017 | 13 | 2017028 | SRR3188272 |
| E2017000745 | 1/9/2017 | 17 | 2017001 | SRR5105390 |
| E2017001788 | 1/29/2017 | 17 | 2017001 | SRR3437476 |
| E2017001956 | 2/4/2017 | 17 |  | SRR4022300 |
| E2017002562 | 2/10/2017 | 13 |  | SRR4039116 |
| I2017002460 | 2/10/2017 | 17 |  | SRR5117053 |
| E2017003603 | 3/5/2017 |  |  | SRR1822278 |
| E2017003833 | 3/7/2017 | 13 | 2017006 | SRR1982152 |
| E2017003821 | 3/7/2017 | 13 | 2017006 | SRR1982234 |
| I2017003687 | 3/10/2017 | 17 | 2017029 | SRR2035434 |
| E2017004214 | 3/14/2017 | 17 | 2017029 | SRR2035407 |
| E2017004322 | 3/17/2017 |  | 2017012 | SRR2056006 |
| I2017004308 | 3/20/2017 | 13 | 2017006 | SRR2056005 |
| E2017004460 | 3/21/2017 | 17 | 2017001 | SRR2072485 |
| E2017004822 | 3/22/2017 | 13 |  | SRR2072492 |
| E2017005549 | 3/22/2017 | 17 | 2017012 | SRR2072493 |
| I2017004541 | 3/26/2017 | 17 | 2017010 | SRR2102400 |
| E2017004996 | 3/27/2017 | 17 | 2017010 | SRR2133340 |
| I2017004794 | 3/28/2017 | 17 | 2017001 | SRR2153212 |
| E2017004945 | 3/29/2017 | 17 | 2017001 | SRR2175356 |
| E2017005289 | 4/5/2017 | 17 | 2017001 | SRR2533504 |
| I2017005299 | 4/8/2017 | 17 | 2017001 | SRR2533500 |
| E2017006005 | 4/14/2017 | 17 | 2017015 | SRR2831445 |
| I2017005906 | 4/15/2017 | 17 | 2017015 | SRR2914507 |
| I2017005940 | 4/17/2017 | 13 | 2017006 | SRR2954253 |
| E2017006068 | 4/17/2017 | 17 | 2017010 | SRR2968059 |
| I2017006023 | 4/18/2017 | 17 |  | SRR2993266 |
| E2017006554 | 4/27/2017 | 13 |  | SRR3217351 |
| E2017006722 | 5/1/2017 | 17 |  | SRR3372232 |
| I2017008662 | 6/3/2017 | 17 |  | SRR3948444 |
| E2017009232 | 6/12/2017 | 13 |  | SRR4175544 |
| I2017009411 | 6/18/2017 | 13 |  | SRR4175563 |
| I2017009966 | 6/27/2017 | 17 | 2017018 | SRR5823590 |
| I2017009925 | 6/27/2017 | 17 | 2017027 | SRR5823615 |
| E2017013009 | 7/24/2017 | 17 |  | SRR6313693 |
| I2017011880 | 7/25/2017 | 17 | 2017018 | SRR6327022 |
| I2017011816 | 7/25/2017 | 17 | 2017027 | SRR6371492 |
| I2017011886 | 7/25/2017 | 17 | 2017027 | SRR3110500 |
| I2017013601 | 8/17/2017 | 17 |  | SRR1982053 |
| I2017013770 | 8/22/2017 | 17 | 2017027 | SRR2533508 |
| E2017016610 | 10/2/2017 |  |  | SRR5382686 |
| I2017016764 | 10/6/2017 | 17 |  | SRR5429822 |
| I2017018384 | 11/2/2017 | 17 |  | SRR5749997 |
| E2017019275 | 11/19/2017 |  |  | SRR5680494 |
| I2017019680 | 11/28/2017 | 17 |  | SRR3110497 |
| E2017020133 | 12/5/2017 | 17 | 2018001 | SRR3151853 |
| E2017020916 | 12/24/2017 | 17 | 2018001 | SRR5440648 |
| E2018000104 | 12/30/2017 | 17 |  | SRR5182244 |
| E2015005174 | 4/10/2015 |  |  | SRR3372230 |
| E2015002922 | 2/19/2015 |  | 2015001 | SRR2106112 |
| I2016017854 | 11/11/2016 |  | 2016033 | SRR3223813 |
| E2015003840 | 3/11/2015 |  |  | SRR2140781 |
| I2016006642 | 5/2/2016 |  |  | SRR5584009 |
| E2017008624 | 5/31/2017 |  |  | SRR3824041 |
| E2017014242 | 8/22/2017 |  |  | SRR2533503 |
| E2017015775 | 9/18/2017 |  |  | SRR4340444 |
| E2015003281 | 2/26/2015 |  |  | SRR2153467 |
| E2015001358 | 1/16/2015 |  |  | SRR1822280 |
| E2015011861 | 8/15/2015 |  |  | SRR6216166 |
| E2015004060 | 3/19/2015 |  |  | SRR2153206 |
| E2016007250 | 5/12/2016 | 18 |  | SRR5583985 |
| E2016008572 | 6/4/2016 | 18 |  | SRR6313709 |
| I2016007658 | 5/23/2016 |  |  | SRR5864546 |
| E2017000639 | 1/6/2017 |  |  | SRR2724349 |
| E2017005629 | 4/7/2017 | 19 | 2017009 | SRR2533499 |
| E2017005651 | 4/10/2017 | 19 | 2017009 | SRR2533568 |
| E2017005671 | 4/10/2017 | 19 | 2017009 | SRR2533570 |
| I2017005781 | 4/10/2017 | 19 | 2017009 | SRR2533569 |
| E2017005793 | 4/11/2017 | 19 | 2017009 | SRR2584382 |
| I2017005640 | 4/11/2017 | 19 | 2017009 | SRR2559364 |
| E2017006900 | 5/3/2017 | 19 | 2017009 | SRR3457765 |
| I2017006753 | 5/3/2017 | 19 | 2017009 | SRR3571299 |
| E2017008126 | 5/24/2017 | 19 | 2017009 | SRR3743173 |
| E2017008099 | 5/26/2017 | 19 | 2017009 | SRR3721482 |
| E2015002112 | 2/3/2015 | 20 |  | SRR2082853 |
| E2015004124 | 3/20/2015 |  |  | SRR2968600 |
| E2015004502 | 4/1/2015 | 20 |  | SRR2831447 |
| E2015015187 | 10/18/2015 |  |  | SRR1988493 |
| E2015015239 | 10/20/2015 | 21 |  | SRR1988495 |
| E2015015776 | 10/29/2015 | 21 |  | SRR1988496 |
| E2016000376 | 1/3/2016 |  |  | SRR2559366 |
| E2016000740 | 1/11/2016 | 22 |  | SRR2724298 |
| E2016003746 | 3/9/2016 | 22 | 2016001 | SRR3330237 |
| I2016005045 | 4/2/2016 | 22 | 2016001 | SRR4175545 |
| E2016005190 | 4/4/2016 | 22 | 2016001 | SRR5132015 |
| I2016005133 | 4/5/2016 | 22 | 2016001 | SRR5217787 |
| I2016005854 | 4/20/2016 | 22 | 2016001 | SRR5429818 |
| E2016008184 | 5/13/2016 | 22 | 2016001 | SRR5750011 |
| E2016017246 | 11/1/2016 | 23 | 2016034 | SRR3136813 |
| E2016017882 | 11/12/2016 | 23 |  | SRR5921391 |
| E2016019090 | 12/6/2016 | 23 | 2016034 | SRR5586984 |
| E2016019960 | 12/21/2016 | 23 | 2016034 | SRR2072491 |
| E2017000458 | 1/5/2017 | 23 |  | SRR2533352 |
| I2017002564 | 2/11/2017 | 23 | 2017003 | SRR5360753 |
| I2017002382 | 2/12/2017 | 23 | 2017003 | SRR5584863 |
| I2017013437 | 8/16/2017 |  |  | SRR1828973 |
| I2017017043 | 10/11/2017 |  |  | SRR5486470 |
| I2017017785 | 10/24/2017 | 24 | 2017023 | SRR5486501 |
| E2017018522 | 10/31/2017 | 24 | 2017023 | SRR5659586 |
| I2017018796 | 11/10/2017 | 24 | 2017025 | SRR5823618 |
| E2017019381 | 11/21/2017 | 24 | 2017025 | SRR5680484 |
| I2017019404 | 11/24/2017 | 24 | 2017025 | SRR5864375 |
| E2015010684 | 7/30/2015 |  |  | SRR6050828 |
| I2017015332 | 9/14/2017 |  |  | SRR4175564 |
| E2015001731 | 1/29/2015 |  |  | SRR2062503 |
| E2017005056 | 3/25/2017 |  | 2017013 | SRR2153463 |
| I2017004903 | 4/1/2017 |  |  | SRR2175342 |
| E2016011001 | 7/18/2016 |  |  | SRR5501024 |
| I2017003022 | 2/24/2017 |  | 2017006 | SRR6386383 |
| E2015001405 | 1/17/2015 |  | 2015001 | SRR1980625 |
| E2016001120 | 1/21/2016 | 25 | 2016008 | SRR2759018 |
| E2016003440 | 3/3/2016 | 25 |  | SRR3306270 |
| I2017011595 | 7/21/2017 |  |  | SRR6313710 |
| I2017013954 | 8/25/2017 |  |  | SRR3509909 |
| E2016012152 | 8/7/2016 |  |  | SRR3131111 |
| E2017002927 | 2/13/2017 |  |  | SRR5429817 |
| E2017008174 | 5/23/2017 |  |  | SRR3743171 |
| E2015007276 | 6/3/2015 |  |  | SRR5281994 |
| E2015004402 | 3/23/2015 |  |  | SRR2533495 |
| E2015005618 | 4/20/2015 |  |  | SRR3696037 |
| E2017003240 | 3/1/2017 |  |  | SRR1828980 |
| E2015001564 | 1/24/2015 |  |  | SRR2047360 |
| E2015005835 | 4/29/2015 |  |  | SRR3928751 |
| E2015006858 | 5/19/2015 |  |  | SRR4256616 |
| E2015008009 | 6/12/2015 |  | 2015011 | SRR5360755 |
| E2015008514 | 6/24/2015 |  | 2015031 | SRR5501013 |
| E2015008504 | 6/25/2015 |  |  | SRR5640242 |
| E2015011310 | 8/11/2015 |  |  | SRR6158670 |
| E2015012606 | 9/1/2015 |  |  | SRR3634484 |
| E2015014375 | 10/3/2015 |  | 2015012 | SRR1982124 |
| E2015017439 | 11/30/2015 |  |  | SRR2047359 |
| E2016002349 | 2/13/2016 |  |  | SRR3198633 |
| I2016018188 | 11/23/2016 |  |  | SRR5360983 |
| E2017011524 | 7/19/2017 |  |  | SRR6133783 |
| E2016003725 | 3/8/2016 |  |  | SRR3372224 |
| E2016003976 | 3/16/2016 |  |  | SRR3372229 |
| I2016004355 | 3/23/2016 |  |  | SRR3400299 |
| E2017005256 | 4/6/2017 |  | 2017011 | SRR2533509 |
| E2016005372 | 4/9/2016 | 26 | 2016012 | SRR5221486 |
| I2016005745 | 4/14/2016 | 26 | 2016012 | SRR5501000 |
| E2016005761 | 4/16/2016 | 26 | 2016012 | SRR5583926 |
| E2016006269 | 4/23/2016 | 26 | 2016012 | SRR5501018 |
| E2016006515 | 4/28/2016 | 26 | 2016012 | SRR5583931 |
| I2016008662 | 6/7/2016 |  |  | SRR6353859 |
| E2016011589 | 7/29/2016 |  |  | SRR1811675 |
| E2016011924 | 8/2/2016 |  | 2016019 | SRR2906170 |
| E2016018931 | 12/4/2016 |  |  | SRR2035428 |
| I2017002484 | 2/13/2017 |  |  | SRR5659637 |
| I2017002732 | 2/13/2017 |  | 2017011 | SRR5750003 |
| E2017003849 | 3/11/2017 |  | 2017013 | SRR2035430 |
| E2017004521 | 3/22/2017 |  |  | SRR2072490 |
| E2017007371 | 5/10/2017 |  |  | SRR3465470 |
| I2017008692 | 6/2/2017 |  |  | SRR3824039 |
| I2017011987 | 7/28/2017 |  |  | SRR3136814 |
| I2017013567 | 8/20/2017 |  |  | SRR2005756 |
| E2017011576 | 7/19/2017 |  |  | SRR6221662 |
| I2017019729 | 11/29/2017 |  |  | SRR3136812 |
| I2017016070 | 9/26/2017 | 27 |  | SRR5366228 |
| I2017016548 | 9/28/2017 | 27 |  | SRR5364219 |
| E2015000973 | 1/15/2015 | 28 |  | SRR1822275 |
| E2015001040 | 1/17/2015 | 28 |  | SRR1982162 |
| E2015001451 | 1/20/2015 | 29 | 2015003 | SRR1982030 |
| E2015001415 | 1/21/2015 | 29 | 2015003 | SRR2054220 |
| E2015001448 | 1/22/2015 | 28 | 2015004 | SRR2035412 |
| E2015001844 | 2/2/2015 | 28 | 2015004 | SRR2062506 |
| E2015002433 | 2/12/2015 | 28 | 2015005 | SRR2082947 |
| E2015002461 | 2/12/2015 | 28 | 2015005 | SRR2082830 |
| E2015002781 | 2/19/2015 | 28 |  | SRR2106113 |
| E2015003394 | 2/28/2015 | 28 | 2015005 | SRR2125971 |
| E2015003379 | 3/3/2015 | 28 | 2015005 | SRR2153476 |
| E2015004532 | 3/30/2015 |  |  | SRR2831442 |
| E2015005668 | 4/23/2015 | 28 | 2015012 | SRR3824040 |
| E2015005665 | 4/24/2015 | 28 |  | SRR3824034 |
| E2015005864 | 5/1/2015 | 28 | 2015012 | SRR3928753 |
| E2015006110 | 5/7/2015 | 28 | 2015013 | SRR3990988 |
| E2015006131 | 5/7/2015 | 28 | 2015013 | SRR4039117 |
| E2015006130 | 5/8/2015 | 28 | 2015013 | SRR3990990 |
| E2015006278 | 5/11/2015 | 28 |  | SRR4175550 |
| E2015006271 | 5/12/2015 | 30 | 2015017 | SRR4237886 |
| E2015007082 | 5/26/2015 | 28 | 2015012 | SRR4294795 |
| E2015007130 | 5/26/2015 | 28 | 2015012 | SRR4292711 |
| E2015007106 | 5/28/2015 | 28 | 2015020 | SRR4733518 |
| E2015007159 | 5/28/2015 | 30 |  | SRR4733516 |
| E2015007326 | 6/4/2015 | 28 | 2015012 | SRR5281989 |
| E2015007427 | 6/6/2015 | 30 |  | SRR5281993 |
| E2015007484 | 6/8/2015 | 28 |  | SRR5296131 |
| E2015008070 | 6/9/2015 | 28 |  | SRR5296134 |
| E2015007967 | 6/11/2015 | 30 |  | SRR5296127 |
| E2015007956 | 6/11/2015 | 30 | 2015017 | SRR5361145 |
| E2015007987 | 6/11/2015 | 30 | 2015017 | SRR5366195 |
| E2015008141 | 6/17/2015 | 28 |  | SRR5382691 |
| E2015008532 | 6/24/2015 | 28 | 2015012 | SRR6809223 |
| E2015008760 | 7/1/2015 | 28 | 2015012 | SRR5628609 |
| E2015009252 | 7/2/2015 | 28 | 2015020 | SRR5865225 |
| E2015009079 | 7/3/2015 | 28 | 2015021 | SRR5808891 |
| E2015009194 | 7/6/2015 | 28 | 2015029 | SRR5822035 |
| E2015009384 | 7/8/2015 | 30 |  | SRR5921355 |
| E2015009908 | 7/14/2015 | 30 |  | SRR5947663 |
| E2015010255 | 7/22/2015 | 28 |  | SRR6026001 |
| E2015010628 | 7/26/2015 | 28 | 2015012 | SRR6038708 |
| E2015010649 | 7/29/2015 | 28 | 2015021 | SRR6050825 |
| E2015010802 | 7/29/2015 | 28 |  | SRR6050815 |
| E2015010948 | 8/6/2015 | 28 | 2015012 | SRR6107259 |
| E2015011381 | 8/10/2015 | 28 |  | SRR6133771 |
| E2015011528 | 8/13/2015 | 28 | 2015029 | SRR6133767 |
| E2015011355 | 8/13/2015 | 28 |  | SRR6158669 |
| E2015011853 | 8/17/2015 | 28 |  | SRR6300164 |
| E2015012057 | 8/20/2015 | 28 |  | SRR6371501 |
| E2015012162 | 8/24/2015 | 28 |  | SRR6434216 |
| E2015012170 | 8/24/2015 | 30 |  | SRR6452145 |
| E2015012695 | 9/1/2015 | 28 | 2015012 | SRR3948447 |
| E2015012816 | 9/2/2015 | 28 | 2015029 | SRR3948441 |
| E2015012699 | 9/3/2015 | 28 | 2015012 | SRR5440654 |
| E2015013115 | 9/13/2015 | 30 | 2015034 | SRR6107183 |
| E2015013546 | 9/19/2015 | 28 | 2015037 | SRR1773594 |
| E2015013963 | 9/27/2015 | 30 | 2015034 | SRR1828972 |
| E2015015299 | 10/15/2015 | 28 | 2015029 | SRR1988494 |
| E2015014944 | 10/16/2015 | 28 | 2015012 | SRR1988450 |
| E2015015391 | 10/22/2015 | 28 |  | SRR1982031 |
| E2015016457 | 11/10/2015 | 28 | 2015038 | SRR1998961 |
| E2015016768 | 11/18/2015 | 28 | 2015037 | SRR1998974 |
| E2015017264 | 11/24/2015 | 28 | 2015038 | SRR2035441 |
| E2016000204 | 12/30/2015 | 28 |  | SRR2082933 |
| E2016001566 | 1/29/2016 | 31 |  | SRR3173544 |
| E2016002209 | 2/8/2016 | 31 |  | SRR3173547 |
| E2016003173 | 2/24/2016 | 28 |  | SRR3223809 |
| I2016004512 | 3/24/2016 | 28 |  | SRR3465460 |
| E2016004542 | 3/25/2016 | 28 | 2016011 | SRR3509910 |
| E2016005025 | 4/1/2016 |  | 2016011 | SRR4022298 |
| E2016005476 | 4/11/2016 | 28 | 2016011 | SRR5296122 |
| E2016005766 | 4/12/2016 | 28 |  | SRR5364218 |
| E2016005675 | 4/14/2016 | 28 | 2016011 | SRR5408955 |
| E2016006274 | 4/24/2016 | 28 | 2016019 | SRR5440659 |
| I2016006669 | 5/4/2016 |  |  | SRR5584633 |
| E2016007926 | 5/24/2016 | 28 |  | SRR5978575 |
| I2016008250 | 5/30/2016 | 28 | 2016019 | SRR6019673 |
| I2016008288 | 6/1/2016 |  |  | SRR6169245 |
| I2016008661 | 6/9/2016 | 28 | 2016022 | SRR6452146 |
| E2016009047 | 6/11/2016 | 28 |  | SRR1998943 |
| I2016009149 | 6/15/2016 | 28 | 2016022 | SRR2035429 |
| E2016009720 | 6/22/2016 | 28 |  | SRR3571305 |
| E2016009735 | 6/24/2016 | 28 |  | SRR6133763 |
| E2016009919 | 6/28/2016 | 28 |  | SRR3135177 |
| E2016010179 | 6/29/2016 | 28 |  | SRR1982120 |
| E2016010234 | 7/4/2016 | 28 |  | SRR5260023 |
| I2016011284 | 7/25/2016 | 28 |  | SRR5590277 |
| I2016011407 | 7/28/2016 | 28 | 2016019 | SRR5680482 |
| E2016011581 | 7/30/2016 |  |  | SRR1982202 |
| I2016012340 | 8/8/2016 | 28 | 2016019 | SRR3271879 |
| E2016012283 | 8/9/2016 | 28 | 2016024 | SRR3400297 |
| I2016012753 | 8/11/2016 |  | 2016019 | SRR3400300 |
| I2016012750 | 8/16/2016 | 28 | 2016024 | SRR3696033 |
| E2016013100 | 8/18/2016 | 28 |  | SRR5182260 |
| E2016014437 | 9/10/2016 | 28 |  | SRR6434141 |
| E2016015840 | 10/3/2016 | 28 | 2016032 | SRR5501030 |
| E2016018645 | 11/28/2016 | 28 | 2016032 | SRR2062505 |
| E2017001443 | 1/20/2017 | 28 |  | SRR3306269 |
| I2017003610 | 3/3/2017 | 28 |  | SRR1811628 |
| I2017003937 | 3/16/2017 | 28 |  | SRR2056004 |
| E2017006030 | 4/13/2017 | 28 |  | SRR2724297 |
| E2017006309 | 4/21/2017 | 28 |  | SRR3151856 |
| I2017008499 | 6/2/2017 | 28 | 2017017 | SRR3948442 |
| E2017008865 | 6/4/2017 | 28 | 2017017 | SRR4022297 |
| E2017009633 | 6/18/2017 | 28 |  | SRR4292712 |
| I2017009636 | 6/20/2017 | 28 | 2017017 | SRR5296124 |
| I2017009758 | 6/21/2017 | 28 |  | SRR5583952 |
| E2017009899 | 6/23/2017 | 28 | 2017017 | SRR5583936 |
| E2017011379 | 7/9/2017 | 28 | 2017017 | SRR5908220 |
| I2017011377 | 7/13/2017 | 28 | 2017017 | SRR5996836 |
| I2017012992 | 8/11/2017 | 28 | 2017017 | SRR3509919 |
| E2017015024 | 9/7/2017 | 28 |  | SRR4175548 |
| I2017015941 | 9/22/2017 | 28 | 2017017 | SRR5249643 |
| E2017017184 | 9/24/2017 | 28 |  | SRR5364217 |
| E2017017628 | 10/4/2017 | 28 |  | SRR5486494 |
| I2017017189 | 10/9/2017 | 28 | 2017017 | SRR5440663 |
| I2017018246 | 10/29/2017 | 28 |  | SRR5645419 |
| E2017018587 | 11/2/2017 | 28 | 2017017 | SRR5749994 |
| I2017018544 | 11/6/2017 | 28 | 2017017 | SRR5851107 |
| E2017018787 | 11/10/2017 | 28 |  | SRR5876320 |
| E2017019437 | 11/18/2017 | 28 | 2017017 | SRR6203427 |
| E2015018531 | 12/20/2015 | 32 | 2016002 | SRR2072483 |
| E2016001421 | 1/24/2016 | 32 | 2016002 | SRR2993268 |
| E2016004517 | 3/22/2016 |  | 2016002 | SRR3400298 |
| E2016004805 | 3/30/2016 | 33 | 2016002 | SRR3660132 |
| E2016005931 | 4/21/2016 | 33 | 2016002 | SRR5440657 |
| I2016006553 | 4/29/2016 | 33 | 2016002 | SRR5501022 |
| E2016012184 | 8/6/2016 |  |  | SRR3110335 |
| E2015001380 | 1/20/2015 | 34 |  | SRR1982128 |
| E2015001699 | 1/27/2015 | 34 |  | SRR2056003 |
| E2015003064 | 2/24/2015 | 34 |  | SRR2140779 |
| E2015004448 | 3/29/2015 |  |  | SRR2559365 |
| E2015005043 | 4/9/2015 | 34 |  | SRR3210397 |
| E2015005063 | 4/10/2015 |  |  | SRR3437475 |
| E2015008421 | 6/17/2015 | 35 |  | SRR5408947 |
| E2015009382 | 7/7/2015 | 35 | 2015031 | SRR5822054 |
| E2015011377 | 8/14/2015 | 35 | 2015030 | SRR6203394 |
| E2015011730 | 8/16/2015 | 35 | 2015030 | SRR6330497 |
| E2015012226 | 8/25/2015 |  |  | SRR6457614 |
| E2015017609 | 12/2/2015 |  |  | SRR2054137 |
| I2016003617 | 3/3/2016 | 36 |  | SRR3330235 |
| I2016004507 | 3/24/2016 | 36 | 2016015 | SRR3457764 |
| E2016005959 | 4/19/2016 | 36 | 2016015 | SRR5429820 |
| E2016006522 | 4/27/2016 | 36 | 2016015 | SRR5501012 |
| E2016006813 | 5/5/2016 | 36 | 2016015 | SRR5584598 |
| I2016007589 | 5/22/2016 | 36 |  | SRR5864555 |
| E2016008726 | 6/8/2016 | 36 |  | SRR6434213 |
| E2016008707 | 6/9/2016 | 36 |  | SRR6485186 |
| E2016009771 | 6/23/2016 | 36 | 2016021 | SRR6026009 |
| E2016012926 | 8/15/2016 | 36 | 2016027 | SRR3490027 |
| E2016012846 | 8/17/2016 | 36 | 2016021 | SRR5032261 |
| E2016012907 | 8/18/2016 | 36 | 2016026 | SRR5182247 |
| E2016013470 | 8/26/2016 | 36 | 2016026 | SRR5221474 |
| E2016013656 | 8/27/2016 | 36 | 2016027 | SRR5360754 |
| I2016014963 | 9/20/2016 | 36 | 2016021 | SRR6133772 |
| E2016015785 | 10/1/2016 | 36 |  | SRR3662623 |
| I2016016786 | 10/25/2016 | 36 |  | SRR5364223 |
| E2017000858 | 1/16/2017 | 37 |  | SRR3271877 |
| E2017003039 | 2/22/2017 | 37 | 2017004 | SRR6386391 |
| I2017002955 | 2/22/2017 | 37 | 2017004 | SRR6386389 |
| E2017003065 | 2/26/2017 | 37 | 2017004 | SRR1773636 |
| E2017003216 | 3/2/2017 | 37 | 2017004 | SRR1828986 |
| E2017003613 | 3/4/2017 | 37 | 2017004 | SRR1813444 |
| I2017003237 | 3/6/2017 | 37 |  | SRR1980609 |
| I2017004331 | 3/20/2017 | 37 | 2017004 | SRR2062502 |
| I2017004375 | 3/21/2017 | 37 | 2017004 | SRR2072484 |
| I2017004390 | 3/21/2017 | 37 | 2017004 | SRR2082848 |
| I2017004431 | 3/22/2017 | 37 | 2017004 | SRR2082940 |
| I2017004432 | 3/23/2017 | 37 | 2017004 | SRR2106106 |
| I2017004802 | 3/26/2017 | 37 | 2017004 | SRR2106117 |
| E2017004897 | 3/28/2017 | 37 |  | SRR2153207 |
| E2017005164 | 4/3/2017 |  |  | SRR2533505 |
| I2017005033 | 4/3/2017 | 37 | 2017004 | SRR2533506 |
| I2017005253 | 4/8/2017 | 37 |  | SRR2533501 |
| I2017007457 | 5/15/2017 | 37 | 2017004 | SRR3490013 |
| E2017008058 | 5/22/2017 | 37 | 2017004 | SRR3509923 |
| E2017008458 | 5/30/2017 | 37 |  | SRR3824044 |
| E2017008673 | 6/1/2017 | 37 |  | SRR3824033 |
| I2017009520 | 6/19/2017 | 37 |  | SRR4429090 |
| I2017009764 | 6/23/2017 | 37 |  | SRR5605896 |
| E2017010770 | 7/8/2017 | 37 |  | SRR5908207 |
| E2017015248 | 9/11/2017 | 38 | 2017022 | SRR4175546 |
| E2017016566 | 9/29/2017 | 38 | 2017022 | SRR5408968 |
| E2017016979 | 10/5/2017 | 38 | 2017022 | SRR5429828 |
| E2017008150 | 5/23/2017 | 39 | 2017016 | SRR3634505 |
| E2017008152 | 5/25/2017 | 39 | 2017016 | SRR3707418 |
| E2017010519 | 7/3/2017 | 39 |  | SRR5851120 |
| E2015004512 | 4/2/2015 |  |  | SRR2993265 |
| E2016000215 | 1/1/2016 | 40 |  | SRR2584383 |
| E2016001099 | 1/20/2016 | 40 | 2016003 | SRR2724348 |
| E2016001477 | 1/26/2016 | 40 | 2016003 | SRR3110337 |
| E2016004753 | 3/26/2016 | 40 | 2016003 | SRR3571300 |
| E2017004452 | 3/20/2017 | 41 | 2017014 | SRR2062507 |
| E2017004814 | 3/27/2017 | 41 | 2017014 | SRR2140786 |
| E2015014906 | 10/13/2015 |  |  | SRR1988508 |
| E2016019994 | 12/21/2016 |  |  | SRR2096593 |
